# Effect of Isomeric Amine Chain Extenders and Crosslink Density on the Properties of Liquid Crystal Elastomers

**DOI:** 10.3390/ma13143094

**Published:** 2020-07-10

**Authors:** Yoojin Lee, Subi Choi, Beom-Goo Kang, Suk-kyun Ahn

**Affiliations:** 1 Department of Polymer Science and Engineering, Pusan National University, Busan 46241, Korea; marvjoanne897@gmail.com (Y.L.); subi.choii@gmail.com (S.C.); 2 Department of Chemical Engineering, Soongsil University, Seoul 06978, Korea

**Keywords:** liquid crystal elastomer, actuation, surface alignment, structure-property relationship

## Abstract

Among the various types of shape changing materials, liquid crystal elastomers (LCEs) have received significant attention as they can undergo programmed and reversible shape transformations. The molecular engineering of LCEs is the key to manipulating their phase transition, mechanical properties, and actuation performance. In this work, LCEs containing three different types of butyl groups (n-, iso-, and sec-butyl) in the side chain were synthesized, and the effect of isomeric amine chain extenders on the thermal, mechanical, and actuation properties of the resulting LCEs was investigated. Because of the considerably low reactivity of the sec-butyl group toward the diacrylate in the LC monomer, only a densely crosslinked LCE was synthesized. Most interestingly, the mechanical properties, actuation temperature, and blocking stress of the LCEs comprising isobutyl groups were higher than those of the LCEs comprising n-butyl groups. This difference was attributed to the presence of branches in the LCEs with isobutyl groups, which resulted in a tighter molecular packing and reduced the free volume. Our results suggest a facile and effective method for synthesizing LCEs with tailored mechanical and actuation properties by the choice of chain extenders, which may advance the development of soft actuators for a variety of applications in aerospace, medicine, and optics.

## 1. Introduction

Liquid crystal elastomers (LCEs), which consist of lightly crosslinked networks of mesogenic units, are a distinct class of shape changing materials. In LCEs, anisotropic mesogenic units allow the formation of diverse self-organized mesophases, while loosely crosslinked chain networks allow a substantial change in the LC ordering and entropy elasticity [1,2,3,4]. LCEs have attracted significant research interest, as they can undergo large and programmed shape transformations in a reversible manner under various external stimuli that can disrupt LC ordering, thereby inducing a significant conformational change in the polymeric chains [3]. In addition, the shape change of LCEs can be effectively manipulated by a spatial control of the director profile that enables complex shape changes even when the LCE is compositionally homogenous [5,6]. These unique characteristics of LCEs promote their utilization as interesting components for developing various types of smart materials and systems, including artificial muscles [7,8], soft robots [9,10], smart coatings [11], and optical [12] and biomedical devices [13].

Several synthetic strategies have been developed for LCEs so far. Classical LCE synthesis, pioneered by Finkelmann et al., employs hydrosilylation chemistry to graft vinyl-functionalized mesogens and crosslinkers onto polyhydrosiloxane to prepare side-chain LCEs [14]. Hydrosilylation chemistry was also applied to prepare main-chain LCEs by chain-extending a divinyl LC monomer with a disiloxane chain extender and subsequently crosslinking with a tetrasiloxane crosslinker [15]. Controlled polymerization methods such as atom transfer radical polymerization [16], reversible addition-fragmentation chain transfer polymerization [17], and ring-opening metathesis polymerization [18] have also been exploited to prepare well-defined side-chain LC copolymers, whose subsequent crosslinking yielded side-chain LCEs. More recently, extensive studies on LCE synthesis have been reported based on diacrylate-, divinyl-, or diglycidyl-functionalized LC monomers (so-called reactive mesogens). These monomers can first be chain-extended using alkyl dithiols [19,20,21,22,23,24,25,26,27,28], alkyl amines [29,30,31], or alkyl dicarboxylic acids [32,33], and then they can be crosslinked in the presence or absence of multifunctional crosslinkers to obtain main-chain LCEs. One of the major advantages of the above-mentioned method is that various LC alignment methods can be integrated during LCE synthesis.

As previously mentioned, reversible actuation is the most intriguing feature of LCEs, and various factors affect the actuation performance (e.g., work capacity, actuation strain, actuation rate, actuation temperature, and hysteresis). One of the keys that affect the actuation performance is thermal and mechanical properties of intrinsic LCEs. Therefore, several strategies have been explored to tailor the network structure of LCEs, thus manipulating the thermal transitions as well as the mechanical and actuation properties. For example, a high crosslink density increases the glass transition temperature and mechanical stiffness of LCEs; however, the work capacity decreases due to a low actuation strain [34,35,36]. As separately demonstrated by the research groups of Ware [37] and Yakacki [38], crystallizable LCEs not only increase the mechanical properties, including stiffness and toughness, but also enhance the blocking stress and work capacity of thiol-acrylate LCEs. Recently, our group also reported that the glass transition temperature and mechanical properties of main-chain LCEs can be effectively manipulated by adjusting the length of the alkyl groups in the primary amine chain extenders, which can systematically alter the actuation temperature [30]. Based on interpenetrating LCE architectures, Yang et al. achieved enormous blocking stress (2.53 MPa) and work capacity (1267.7 kJ/m^3^), which are 7 and 31 times larger than those of skeletal muscles (0.35 MPa and 40 kJ/m^3^), respectively. Such impressive actuation performance was attributed to extremely strong mechanical properties originating from fully miscible interpenetrating networks that provide both chemical and physical networks, and the ability to preserve chain anisotropy even at high temperatures [39]. As a slightly different approach, White’s research group demonstrated that a thick, laminated LCE film with complex director profiles can significantly improve force output and stroke [40].

In this study, we efficiently tailored the thermal, viscoelastic, and mechanical properties, and thus the resulting actuation performance, of a main-chain LCE via a molecular engineering approach. A series of LCEs was synthesized by chain-extending diacrylate-functionalized nematic monomers by aza-Michael addition to obtain LC oligomers (LCOs), which were then photocrosslinked to produce LCEs. In particular, we examined three butylamine chain extenders that are primary amines but have different alkyl groups, namely n-butylamine (n-BA), isobutylamine (iso-BA), and sec-butylamine (sec-BA). Depending on the isomer of butylamines, the reactivity toward aza-Michael addition was considerably different, which was caused by different steric hindrances. For example, the nematic monomer could not be effectively chain-extended with sec-BA by simple heating and resulted in a low-molar-mass LCO, thus producing a LCE with a high crosslink density. Most interestingly, the LCEs containing the isobutyl group exhibited higher glass transition temperature, mechanical properties, actuation temperature, and blocking stress than the LCEs containing the n-butyl group and having comparable crosslink density. The difference was attributed to the presence of branches in the LCEs, which probably led to tighter molecular packing and reduced the free volume. To the best of our knowledge, our study is the first systematic investigation on the effect of isomeric chain extenders on the thermal, mechanical, and actuation properties of LCEs and can improve the fundamental understanding of the structure–property relationship of LCEs for next-generation actuating materials.

## 2. Materials and Methods

### 2.1. Materials

1,4-Bis [4-(6-acryloyloxyhexyloxy)benzoyloxy]-2-methylbenzene (RM82) was purchased from Synthon Chemicals (Bitterfeld-Wolfen, Germany). n-BA, iso-BA, and sec-BA were purchased from Thermo Fisher Scientific (Waltham, MA, USA). Irgacure-369 (I-369) was donated by the BASF Corporation. All chemicals were used without further purification.

### 2.2. Fabrication of Cells

Surface-aligned glass cells were fabricated to prepare planarly aligned LCEs. Two glass slides were cleaned with acetone and isopropanol by bath sonication and then treated with oxygen plasma for 10 min. Then, the glass slides were coated with Elvamide solution (0.125 wt % in methanol) using a spin coater and rubbed with a velvet cloth. The rubbed glass slides were assembled in an anti-parallel configuration separated by spacers (50 or 100 μm).

### 2.3. Synthesis of Surface-Aligned Liquid Crystal Elastomers

The LC monomer (RM82), amine chain extender (n-BA, iso-BA, or sec-BA), and I-369 were added together in a 5 mL vial. The molar ratio between LC monomer and chain extender was adjusted to produce LCOs with nearly the same degree of polymerization (DP), while the amount of I-369 was fixed as 2 wt % in the LC mixture. The molar ratios of LC monomer to chain extender for each sample are listed in Table 1. After heating and vigorous vortexing, the LC mixture was filled into the surface-aligned glass cells at 80 °C (nematic temperature), and the cells were placed in an oven (80 °C) for 24 h to allow the step-growth oligomerization to proceed via aza-Michael addition. Then, the cells containing LCOs were exposed to UV light (OmniCure S1500, λ = 365 nm and 30 mW/cm^2^) (Excelitas, Waltham, MA, USA) for 30 min for photopolymerization to obtain monodomain LCEs.

### 2.4. Synthesis of Polydomain Liquid Crystal Elastomers

Polydomain LCEs, in which the nematic directors of individual domains are randomly oriented, were synthesized by compression molding the LCOs; they were placed between two glass slides separated by a 50 μm spacer at 110 °C (isotropic temperature) using a mini hot press (Specac Mini Pellet Press). Then, the glass cell containing the LCO was moved to a hot plate (85 °C, nematic temperature) and exposed to UV light (λ = 365 nm and 30 mW/cm^2^) for 30 min.

### 2.5. Gel Fraction

The LCEs were placed in a 20 mL vial and immersed in chloroform for 48 h without stirring to extract the uncrosslinked part of the LCEs. Then, the LCEs were vacuum-dried for 24 h at room temperature. The mass of the sample before and after extraction was measured, and the gel fraction (%) was determined by the following equation:

(1) $$\mathrm{Gel}\;\mathrm{fraction}\;\left(\%\right)=\frac{m_{f}\;\left(mg\right)}{m_{i}\;\left(mg\right)}\times 100\%$$

where *m_i_* is the initial mass of the LCE before extraction, and *m_f_* is the final dried mass of the LCE after extraction.

### 2.6. Characterization

^1^H NMR spectroscopy was conducted in CDCl_3_ using a 500 MHz Varian spectrophotometer (Palo Alto, CA, USA). The phase transition temperatures and thermal actuation of the LCEs were observed using a polarizing optical microscope (POM, Nikon Eclipse LV100N POL, Tokyo, Japan) equipped with a heating stage (Linkam LTS420, Tadworth, UK) and ToupView software (version 3.7, ToupTek Photonics, Hangzhou, China). Differential scanning calorimetry (DSC) was performed using a TA Instruments differential scanning calorimeter (Discovery DSC25, New Castle, DE, USA) with a heat/cool/heat protocol under a nitrogen atmosphere. Approximately 3.5 mg of a sample was heated to 150 °C, cooled to −50 °C, and re-heated to 150 °C at a ramp rate of 10 °C/min. The thermal transitions were determined from the second heating cycle using the TRIOS software (version 5.0.0, TA Instruments, New Castle, DE, USA). Thermogravimetric analysis (TGA) was performed using a TA Instruments thermogravimetric analyzer (TGA Q50). Approximately 7.5 mg of a sample was placed in a platinum pan and heated to 800 °C at a heating rate of 10 °C/min. Dynamic mechanical analysis (DMA) was performed to measure the tensile and viscoelastic properties using a dynamic mechanical analyzer (TA instruments Q850) with a tension film clamp. For stress-strain measurements, a rectangular monodomain LCE specimen (L × W × T = 8.0 mm × 4.0 mm × 0.05 mm) was equilibrated at 30 °C under a preload of 0.01 N, and the force was increased at a rate of 0.2 N/min. The viscoelastic properties of the LCEs were investigated by heating the polydomain LCE (L × W × T = 7.0 mm × 5.0 mm × 0.1 mm) from −40 to 120 °C at a ramp rate of 3 °C/min under a frequency of 1 Hz. The maximum peak of the tanδ curve was used to determine the glass transition temperature (T_g_). For isostrain measurements, a monodomain LCE specimen (L × W × T = 8.0 mm × 4.0 mm × 0.1 mm) was heated from 25 to 200 °C with a preload force of 0.01 N and a fixed strain of 0.01%.

## 3. Results and Discussion

### 3.1. Synthesis of LCEs

**Figure 1:**
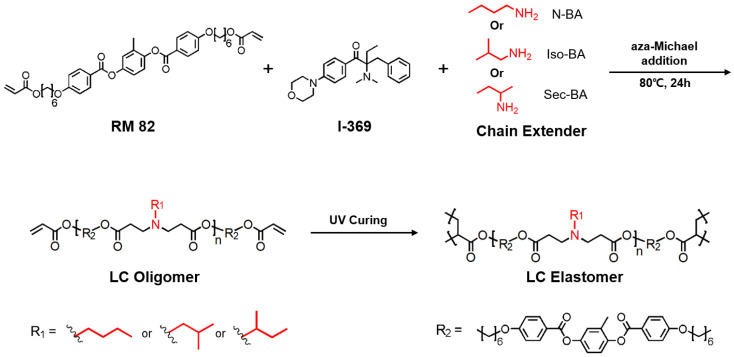
Synthesis of liquid crystal elastomers (LCEs) using three butylamines (n-, iso-, or sec-butylamines) as chain extenders.

Surface-aligned LCEs chain-extended using three different isomeric amines (i.e., n-BA, iso-BA, or sec-BA) were synthesized inside a surface-aligned cell by two orthogonal reactions (i.e., step-growth polymerization and photocrosslinking) by following a previously reported protocol (Figure 1) [29,30]. In the first step, an excess of diacrylate-functionalized LC monomer (RM82) underwent chain-extension by reacting with n-BA, iso-BA, or sec-BA through aza-Michael addition, which yielded diacrylate-functionalized LCOs. Subsequently, the LCOs were exposed to UV light to crosslink the remaining diacrylate groups to produce LCEs. Hereafter, we refer to the LCEs prepared with n-BA as LCE-N1 or LCE-N2, with iso-BA as LCE-I1 or LCE-I2, and with sec-BA as LCE-S1, where 1 and 2 represent high and low crosslink density of the LCE, respectively.

To investigate the effect of isomeric amine chain extenders on the thermal, mechanical, and actuation properties, we prepared a set of LCEs with a similar degree of crosslink density (i.e., a similar molecular weight (MW) of LCOs between the crosslinks). In general, the key to determining the MW of LCOs synthesized by step-growth polymerization is the stoichiometric imbalance between the LC monomer and chain extender [36,40,41]. However, although the amine chain extenders examined in this study were primary amines, they showed different reactivities toward the diacrylate groups in the LC monomer during the aza-Michael addition reaction (i.e., n-butyl > isobutyl > sec-butyl), which is possibly because of the difference in steric hindrance [42]. Considering the different reactivities, the stoichiometry of LC monomer to chain extender was carefully adjusted to be 1.81:1 for LCE-N1, 1.33:1 for LCE-N2, 1.47:1 for LCE-I1, 1.03:1 for LCE-I2, and 1.01:1 for LCE-S1. In other words, the stoichiometric imbalance between the LC monomer and amine chain extender in the feed was higher for LCE-N than for LCE-I and LCE-S.

### 3.2. Molecular Characterization

**Figure 2:**
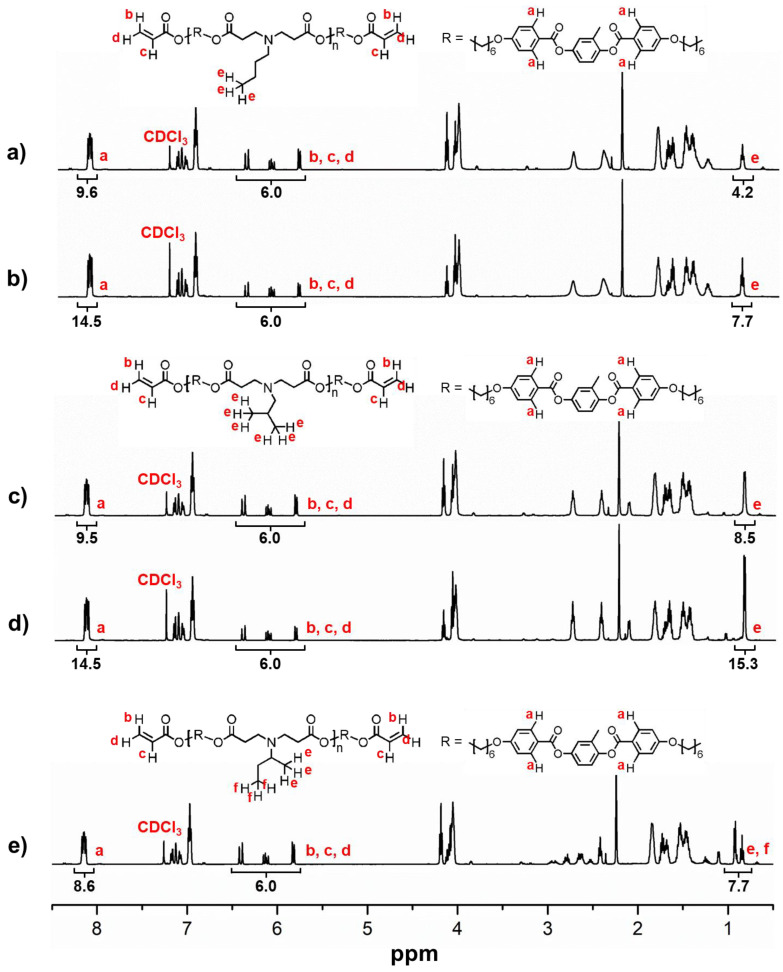
^1^H NMR spectra of LC oligomers (LCOs) in CDCl_3_: (**a**) LCO-N1, (**b**) LCO-N2, (**c**) LCO-I1, (**d**) LCO-I2, and (**e**) LCO-S1. The protons used for calculating the degree of polymerization (DP) of the LCOs are marked in red.

To determine the DP and number average molecular weight (M_n_) of the LCOs, end-group analysis was performed by ^1^H NMR spectroscopy. The ^1^H NMR spectra of LCE-N1, LCE-N2, LCE-I1, LCE-I2, and LCE-S1 and the integration values used to calculate the DP are shown in Figure 2. The M_n_ values of the LCOs were determined by a protocol similar to that described in our previous report [40], and the values are listed in Table 1. Briefly, peaks b (6.40 ppm), c (6.13 ppm), and d (5.82 ppm) correspond to the six protons in the diacrylate end groups in the LCOs, while peak a (8.15 ppm) corresponds to the four aromatic protons in the repeating units. Based on the ratio of the three peaks (b, c, and d) to peak a, the DP and the corresponding M_n_ of the LCOs were calculated as follows: DP = 1.4 (M_n_ = 1720 g/mol) for LCE-N1, DP = 2.6 (M_n_ = 2610 g/mol) for LCE-N2, DP = 1.4 (M_n_ = 1720 g/mol) for LCE-I1, DP = 2.6 (M_n_ = 2610 g/mol) for LCE-I2, and DP = 1.2 (M_n_ = 1570 g/mol) for LCE-S1. The integration values of peak e and peak f corresponding to the protons in the methyl (CH_3_) groups on the side chains can be also used to determine the DP. On the one hand, the DP of LCO-S1 was less than 1.2, even when the molar ratio of LC monomer to sec-BA was 1.01, which indicates the poor reactivity of sec-BA. On the other hand, the higher reactivity of n-BA and iso-BA led to an increase in the DPs of LCO-N1 and LCO-I1 to over 1.2. Since a DP of 1.2 is not sufficiently large to produce a true elastomeric network, we synthesized two additional LCOs (LCO-N2 and LCO-I2) with a higher DP (2.6). Because of the MW limitation of LCE-S, we focused more on the properties of LCE-N and LCE-I.

**Figure 3:**
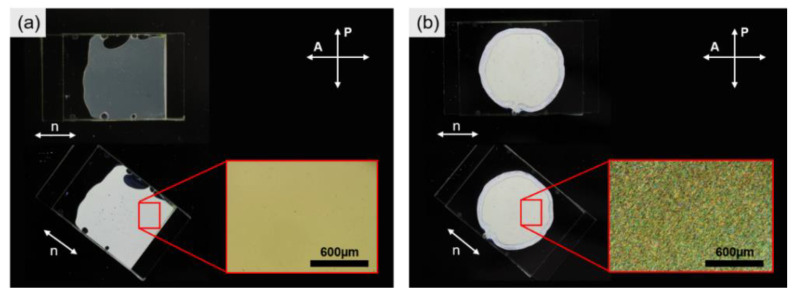
Alignment of LCEs observed between the crossed polarizers and under a POM (inset): (**a**) monodomain LCE and (**b**) polydomain LCE.

For various characterizations, LCEs with two different orientations (monodomain and polydomain) were prepared. The digital and POM images of monodomain and polydomain LCE-N1 between cross polarizers are shown in Figure 3. As expected, monodomain LCE-N1 appeared the brightest when oriented at 45° relative to the crossed polarizers, while polydomain LCE did not exhibit angle-dependent changes in its brightness.

### 3.3. Thermal, Viscoelastic, and Mechanical Properties

The nematic-isotropic phase transition temperatures (T_ni_) of the LC mixtures and LCOs were determined under POM while cooling from the isotropic phase, and the values are listed in Table 1. The T_ni_ of the LCOs were slightly higher than those of the LC mixtures. However, the T_ni_ of the LC mixtures (and LCOs) prepared with different isomeric amines were not much different as long as their DPs were comparable.

**Figure 4:**
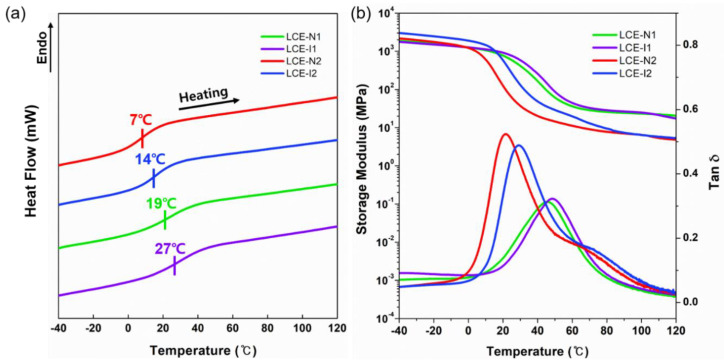
(**a**) DSC thermograms of LCEs during second heating cycle at a heating rate of 10 °C/min. (**b**) Viscoelastic properties of polydomain LCEs.

The T_g_ of the LCEs were determined from the second heating curves of DSC (Figure 4a and Table 1). Among the LCEs with a low DP (i.e., LCE-N1, LCE-I1, and LCE-S1), the T_g_ of LCE-N1 was lower than those of LCE-I1 and LCE-S1. Similarly, the T_g_ of LCE-N2 was lower than that of LCE-I2. The results suggest that the chain mobility of the LCE decreased when the linear alkyl group was replaced with the branched alkyl group. Our observation is consistent with the trend of the T_g_ of conventional polyacrylates with different types alkyl side chains [43]. Furthermore, the T_g_’s of LCE-N2 and LCE-I2 were lower than those of LCE-N1 and LCE-I1. This is because of the higher MW between the crosslinks of LCEs, which resulted in a lower crosslink density [36,41]. Thermal degradation temperature (T_d_) was also measured by TGA to evaluate the thermal stability of LCEs (Table 1). In general, the T_d_ of LCEs under a nitrogen environment is higher than 270 °C. Similar to the T_g_, the T_d_’s of the lightly crosslinked LCEs (LCE-N2 and LCE-I2) were lower than those of densely crosslinked LCE; this implies that the crosslink density can also affect the thermal stability of LCEs. The gel fractions (G) of all the LCEs were greater than 89%, which indicates sufficient crosslinking in the LCEs (Table 1). Based on the comparable DPs of LC oligomers and gel fractions of the LCEs examined in this study, the thermal and mechanical properties of the LCEs (i.e., LCE-N1 vs. LCE-I1, and LCE-N2 vs. LCE-I2) are attributed to the difference in the molecular structures of the LCEs.

The viscoelastic properties of polydomain LCEs (LCE-N1, N2, I1, and I2) were determined by DMA (Figure 4b). As determined from the maximum peak of the tan δ curve, glass transitions occurred at 46, 48, 22, and 29 °C for LCE-N1, LCE-I1, LCE-N2, and LCE-I2, respectively. These values are higher than those determined by DSC measurements and can be attributed to the larger sample dimensions required for DMA, which cause a slower heat transfer to the sample, and the different measurement conditions of DSC (static) and DMA (dynamic). The T_g_’s of the LCEs with a high crosslink density (LCE-N1 and LCE-I1) were higher than those of the LCEs with a low crosslink density (LCE-N2 and LCE-I2). In addition, the T_g_’s of the LCEs chain-extended with iso-BA were slightly higher than those of the LCEs chain-extended with n-BA. These results are in good agreement with the DSC results. Furthermore, the tan δ peak values were approximately 1.6 times greater than those of the LCEs with a high crosslink density, which implies that these samples have higher damping capabilities compared with the LCEs having a higher crosslink density [25].

Above the glass transition region, all the LCEs exhibited rubbery plateaus, which indicate the successful formation of a chain network. The rubbery moduli of LCE-N1 and LCE-I1 were higher than those of LCE-N2 and LCE-I2, which suggest the presence of denser chain networks in these samples. The crosslink densities of the LCEs were calculated by the following equation [44]:

ν_e_ = E’/3RT (2)

where ν_e_ is the crosslink density, E’ is the storage modulus at 100 °C (i.e., at the rubbery plateau), R is the gas constant, and T is the temperature in Kelvin. The crosslink densities of LCE-N1, LCE-I1, LCE-N2, and LCE-I2 were 2.5 × 10^−3^, 2.6 × 10^−3^, 6.8 × 10^−4^, and 6.8 × 10^−4^ mol cm^−3^, respectively.

**Figure 5:**
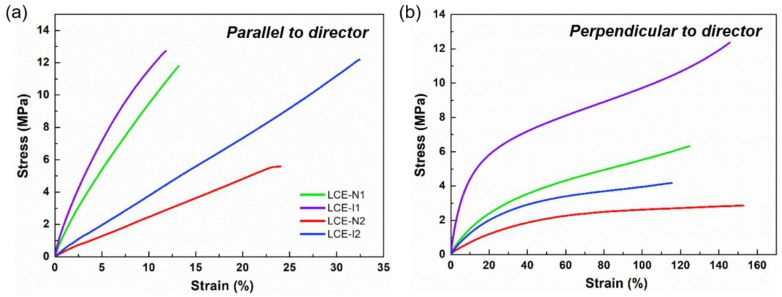
Stress-strain curves of LCEs. Tensile stress was applied (**a**) along the director and (**b**) perpendicular to the director of the LCEs.

The mechanical properties of planarly aligned LCEs (monodomain LCEs) were further investigated by stress-strain analysis by performing DMA in the tensile mode. The stress-strain curves are shown in Figure 5, and the corresponding tensile elastic modulus values are listed in Table 1. Because monodomain LCEs are mechanically anisotropic, the specimens were stretched in two different directions with respect to the director. The LCEs exhibited relatively higher elastic modulus when stretched along the director (Figure 5a) but became compliant when stretched perpendicular to the director (Figure 5b). The soft behavior of the LCEs when stretched perpendicular to the director is because of the reorientation of the director along the stretching direction that allows the LCE to elongate considerably [1]. Consistent with the DSC and viscoelastic measurement results, the LCEs became stiffer (higher elastic modulus) and mechanically stronger (higher tensile strength) with increasing crosslink density and when chain-extended with iso-BA. The investigations on thermal, viscoelastic, and mechanical properties suggest that the properties of LCEs can be effectively manipulated by not only adjusting the crosslink density but also changing the type of isomer of the amine chain extenders.

### 3.4. Thermal Actuation Properties

**Figure 6:**
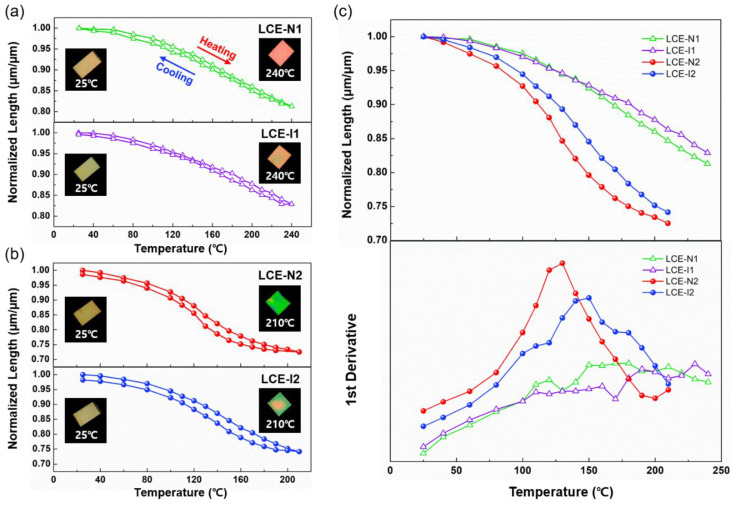
Reversible thermal actuation of planarly aligned LCEs (changes in length during second heating and cooling observed under a POM): (**a**) LCE-N1 (top) and LCE-I1 (bottom); (**b**) LCE-N2 (top) and LCE-I2 (bottom). (**c**) Comparison of thermal actuation of LCEs during second heating.

A salient feature of LCEs is reversible shape change, particularly through nematic-isotropic phase transition. We examined the thermal actuation properties of the LCEs by monitoring the dimensional changes of the planarly aligned samples while heating and cooling under POM. It should be noted that the LCEs were heated only up to 240 °C to avoid degradation. As shown in Figure 6a,b, which depicts the second heating-cooling cycles, all the LCEs exhibited excellent reversible shape change (i.e., contraction upon heating and elongation upon cooling).

However, considerable differences were observed in the actuation strain and actuation temperature depending on the type of LCE (Figure 6c). First, the lightly crosslinked LCEs exhibited larger actuation strains (27.5% for LCE-N2 and 25.9% for LCE-I2) compared with the densely crosslinked LCEs (18.7% for LCE-N1 and 17.1% for LCE-I1). Second, the actuation temperatures, evaluated by the first derivative of the change in length with temperature, of the lightly crosslinked LCEs (130 °C for LCE-N2 and 150 °C for LCE-I2) were much lower than those of the densely crosslinked LCEs. In fact, LCE-N1 and LCE-I1 did not show distinct peaks in the first derivative curve of the dimensional change because there was no abrupt dimensional change when heated up to 240 °C. The lower actuation strain and higher actuation temperature of the densely crosslinked LCEs can be attributed to the tight chain networks in LCE-N1 and LCE-I1 that restricted the disruption of the mesogenic order. In contrast, the loose chain networks in the LCEs facilitated larger changes in the mesogenic order, resulting in a lower actuation temperature and higher actuation strain. Lastly, the actuation temperature of the LCEs chain-extended with iso-BA (LCE-I1 and LCE-I2) was slightly higher than that of the corresponding LCEs chain-extended with n-BA (LCE-N1 and LCE-N2). This is probably because the reduced chain mobility in LCE-I1 and LCE-I2 requires a higher thermal input to disrupt the mesogenic ordering, which in turn alters the chain conformations in the LCEs.

**Figure 7:**
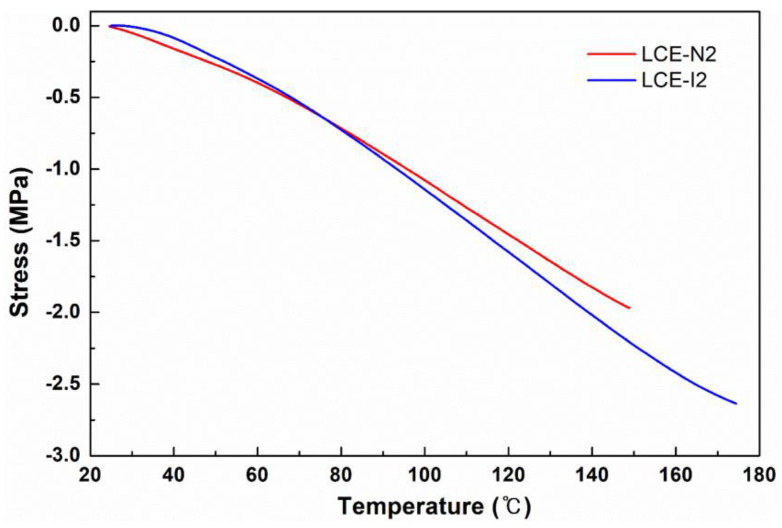
Isostrain test results of planarly aligned LCE-N2 and LCE-I2.

To evaluate the capability of the LCEs to generate stress during thermal actuation, DMA was performed in the isostrain mode to measure the contractile stress. During the test, a constant strain (0.01%) was maintained, and the stress required for maintaining the strain (i.e., blocking stress) was measured as a function of temperature. For this test, the loosely crosslinked monodomain LCEs (i.e., LCE-N2 and LCE-I2) were chosen because of their relatively large dimensional change upon heating compared with the other LCEs. As shown in Figure 7, LCE-N2 broke at 149 °C, generating a blocking stress of 1.97 MPa, while LCE-I2 broke at 174 °C, generating a blocking stress of 2.63 MPa. At the same temperature (149 °C), the blocking stress of LCE-I2 (2.21 MPa) was higher than that of LCE-N2 (1.97 MPa). At comparable crosslink densities, LCE-I2 generated a higher blocking stress and exhibited a higher breaking temperature, which imply that the rigid nature of the isobutyl group makes LCE-I2 mechanically more robust than LCE-N2; this is in good agreement with the stress-strain test results. Meanwhile, the higher blocking stress of our LCEs compared with previously reported LCEs [34,37,45] is possibly because of the relatively high crosslink density (i.e., high rubbery modulus) of our LCEs.

### 3.5. Discussion

Our investigations suggest that the type of isomer in the chain extender can significantly affect the thermal, mechanical, viscoelastic, and actuation properties of LCEs, which can be attributed to the different dynamics of the lateral side groups. For example, Gomez et al. described the motions of protons on the side chains of poly(3-hexylthiophene-2,5-diyl) using spheres of different radii [46]. Based on solid-state NMR study and quasielastic neutron scattering, they discovered that the radius of the mobility of atoms closer to the backbone is suppressed. In another study, Hong et al. examined the effect of the side-chain branch position on the mobility of backbone and the molecular packing structures of poly(3-alkylthiophenes) (P3AT) derivatives with different side chains [47]. It was revealed that moving the branch point closer to the P3AT backbone reduced the side-chain motions and backbone dynamics and resulted in a tighter molecular packing, thus increasing the glass transition and melting temperatures.

**Figure 8:**
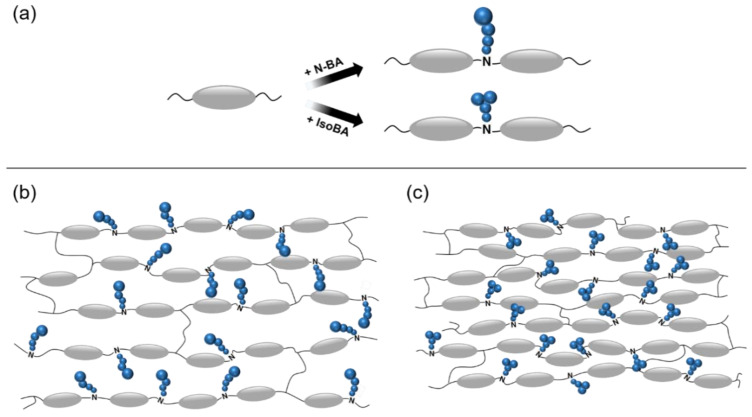
(**a**) Schematic of the diffusion model of side chains in the LCEs. The spheres (blue) represent individual carbon sites, and the ellipsoids (gray) represent the rod-like mesogens. Based on the diffusion model, the molecular packing structures of the (**b**) LCE containing n-butyl group and (**c**) LCE containing isobutyl group are suggested.

Similar to the aforementioned studies, we present a diffusion model of the side chains of LCEs chain-extended with n-BA and iso-BA in Figure 8a, where the size of the spheres represents the range of proton motion, which is expected to increase when the proton is shifted away from the backbone. Therefore, the LCEs chain-extended with the n-butyl group exhibited a greater motion of the side-chain protons. From the perspective of molecular packing predicted in Figure 8b,c, the mesogens in the LCEs chain-extended with the isobutyl group are expected to be more tightly packed (i.e., smaller free volume) than those in the LCEs chain-extended with the n-butyl group because of the presence of a branching point. The lower proton mobility together with tighter molecular packing of the LCEs chain-extended with isobutyl groups would hinder the mobility of the backbone, resulting in higher glass transition temperature and enhanced mechanical properties compared with those of LCEs chain-extended with n-butyl groups.

## 4. Conclusions

Main-chain LCEs chain-extended with various butyl amines (i.e., n-BA, iso-BA, and sec-BA) were synthesized by aza-Michael addition followed by photopolymerization. The reactivities of the amine chain extenders toward the diacrylate groups of the LC monomer were considerably different due to the difference in steric hindrance. Most interestingly, the introduction of the isobutyl group increased the T_g_, mechanical properties, actuation temperature, and blocking stress of the LCEs compared to those of LCEs containing the n-butyl group with a comparable crosslink density. This difference is attributed to the presence of a branch in the isobutyl group, which restricts the motion of backbone and increases molecular packing, thereby decreasing the free volume. In addition, the higher crosslink density of the LCEs not only increased the T_g_, rubbery modulus, mechanical stiffness, and thermal stability, but also increased the actuation temperature and decreased the actuation strain. Our investigation of the structure–property relationship of LCEs based on different types of isomeric chain extenders can improve the understanding of LC-based actuating materials and provide an effective way to tailor their thermal, mechanical, and actuation properties.

## Figures and Tables

**Table 1:** Characterization of materials.

Sample	Acryl/Amine Ratio ^a^	M_n_ (g/mol) ^b^	T_ni_ (°C) ^c^		T_g_ (°C) ^d^	T_d_ (°C) ^e^	E_//_ (MPa) ^f^	E_⊥_ (MPa) ^g^	G (%) ^h^
			LC Mixture	LC Oligomer	LC elastomer				
LCE-N1	1.81	1720	101	106	19	289	173	37	92
LCE-N2	1.33	2610	98	105	7	273	35	10	89
LCE-I1	1.47	1720	100	108	27	299	284	94	91
LCE-I2	1.03	2610	98	106	14	290	43	19	89
LCE-S1	1.01	1570	103	105	23	280	-	-	-

^a^ The molar ratio of LC monomers to chain extenders in the feed. ^b^ Average molecular weight of LC oligomers, as determined by ^1^H NMR spectroscopy. ^c^ Determined by cooling from the isotropic phase under POM. ^d^ T_g_ determined from the second heating curves of DSC. ^e^ Temperature at which 5% weight loss occurs. ^f,g^ Elastic modulus determined from the slope of the stress-strain curves obtained by tensile testing of monodomain LCEs loaded parallel (//) or perpendicular (⊥) to the nematic director. ^h^ Gel fraction values determined by comparing the mass before and after extraction in CHCl_3_.
